# Regarding erector spinae plane block versus caudal block for postoperative analgesia in pediatric patients undergoing inguinal hernia repair: a randomized controlled trial

**DOI:** 10.1080/07853890.2024.2350638

**Published:** 2024-05-11

**Authors:** Cheng-Wen Li, Fu-Shan Xue, Nong He

**Affiliations:** a Department of Anesthesiology, Beijing Friendship Hospital, Capital Medical University, Beijing, People’s Republic of China; b Department of Anesthesiology, Shengli Clinical Medical College of Fujian Medical University, Fujian Provincial Hospital, Fuzhou, China

**To the Editor**

By conducting a randomized controlled trial in 120 pediatric patients who underwent unilateral open inguinal hernia repair, Guan et al. [1] compared the postoperative analgesic efficacy of ultrasound-guided erector spinae plane block (ESPB) and caudal block and demonstrated that ESPB was superior to caudal block in terms of the time to the first rescue analgesia, rescue analgesia requirement, and postoperative pain control. We congratulate the authors for their work that attempted to explore an alternative to caudal block, which is widely regarded as a top choice for multimodal analgesia of pediatric patients undergoing inguinal hernia repair. However, we have several questions on the methodology and results of this study and hope to receive the authors’ reply.

First, for postoperative analgesia, intravenous acetaminophen (15 mg kg^−1^) was administered to all children before the end of surgery. The observed period of this study was 24 h, but it was unclear why acetaminophen was not continued postoperatively according to a scheduled dosing program. There is no consensus on the optimal analgesic combinations, but a multimodal postoperative analgesia regimen of pediatric patients should include the minimum efficacious, safe, and inexpensive non-opioid basic analgesics such as nonsteroidal anti-inflammatory drugs, acetaminophen, and others, unless contraindicated [2]. Furthermore, it is often required that non-opioid basic analgesics should be administered either preoperatively or intraoperatively and continued as per the postoperatively scheduled dosing regime, with reference to a previous study that assessed postoperative analgesic efficacy of local blocks in pediatric patients undergoing elective open inguinal hernia repair [3]. Considering that the perioperative use of non-opioid basic analgesics can significantly improve postoperative pain control and decrease analgesic consumption in pediatric patients [4], we believe that different results would been obtained if this study design included regular application of acetaminophen according to the current recommendations of multimodal postoperative analgesia regimens of pediatric patients [2].

Second, when the postoperative pain score was >3 in the ward, oral ibuprofen 10 mg kg^−1^ was administered as a rescue analgesic in this study. Furthermore, the proportion of patients requiring rescue analgesia was up to 63–97% and was significantly different among the three groups. Considering that the shortest median time to the first rescue analgesia was 1.5 h, we inferred that all rescue analgesics were administered in the ward. We were very interested in knowing whether all patients requiring rescue analgesia only received a single dose of oral ibuprofen during the study period of 24 h after the surgery. Importantly, when used alone, ibuprofen is only a weak basic analgesic that is suitable for the management of mild pain of pain score ≤3 and is hence often used as the cornerstone of multimodal postoperative analgesia regimens in pediatric patients [2]. For moderate or severe postoperative pain in pediatric patients, oral or intravenous opioid analgesics, such as morphine, hydromorphone, and oxycodone, remain the most effective selections [2,5]. We are concerned that an inappropriate choice of rescue analgesic could have increased the risk of rescue analgesia requirements in this study.

Third, when assessing the clinical utility of an intervention intended to improve patient outcomes, the amount of improvement that is important to patients, that is, a minimal clinically important difference, must be considered. The available evidence indicates that the least change of 1.0 on an 11-point pain scale produced by an intervention signifies a clinically important improvement or deterioration [6]. According to the data shown in the Figure 5 of Guan et al.’s article [1], only the difference in pain score at 8-h postoperatively between the ESPB and caudal block groups exceeded the above mentioned minimal clinically important difference. Furthermore, a pain score of ≤3.0 is generally considered adequate postoperative pain control. In fact, median pain scores at all time times postoperatively in the ESPB and caudal block groups were <3. In addition, this study did not report and compare the important outcomes of the current enhanced recovery after surgery practices for pediatric patients, such as the time to first ambulation, time to first flatus, the length of hospital stay, the occurrence of complications, and readmission rate [7]. In these cases, we wonder whether the ESPB, when compared to the caudal block, can improve the postoperative experience and outcomes of pediatric patients undergoing unilateral open inguinal hernia repair.

## Data Availability

Data sharing does not apply to this article as no datasets were generated during the current study.
